# Assistance to black people in the Juquery asylum from 1898 to 1930

**DOI:** 10.11606/s1518-8787.2022056004305

**Published:** 2022-10-07

**Authors:** Amanda Carolina Franciscatto Avezani, João Fernando Marcolan

**Affiliations:** I Universidade Federal de São Paulo Escola Paulista de Enfermagem Programa de Pós-Graduação em Enfermagem São Paulo SP Brasil Universidade Federal de São Paulo. Escola Paulista de Enfermagem. Programa de Pós-Graduação em Enfermagem. São Paulo, SP, Brasil

**Keywords:** Pacientes Internados, Grupo com Ancestrais do Continente Africano, Racismo, Hospitais Psiquiátricos, história, Assistência à Saúde Mental

## Abstract

**OBJECTIVE:**

To reveal the assistance provided to black individuals hospitalized at the Juquery Asylum from 1898 to 1930, having considered the social context and the hegemony of medical knowledge of the time.

**METHODS:**

Exploratory-descriptive, qualitative study, documentary analysis, in medical records of black individuals hospitalized at the Juquery Asylum from 1898 to 1930. The time frame encompassed specific institutional directions as well as the historical, political, economic, and social context experienced by the black population. Held at the archive of the historical and cultural heritage of the Juquery Hospital Complex, between July and December 2019. We used an instrument with questions related to sociodemographic data, date and anamnesis of entry, physical and psychological examination, diagnostic hypothesis, treatments performed, complications, outcome, and motive. The analysis was carried out according to stages of documentary analysis and was based on psychiatric theoretical references of the period.

**RESULT:**

All medical records of the period were read (approximately 6,300), of which about 1,400 were of black individuals. Of these medical records, 457 were included, 140 of women and 317 of men, which were considered to have significant information for the study’s objectives. Most of the participants had long-term hospitalizations, whose motive did not seem to be linked to the possibility of cure or social reintegration, but rather to segregation. From the diagnoses described, the impression is that these subjects composed a niche with immutable, permanent conditions, not amenable to therapeutics that would allow their return to society, exemplified by degeneration. A significant amount of the medical records do not contain data on treatments, which reinforces the hypothesis that they were kept hospitalized not for the purpose of care, but as a deposit of incurability; when they do bring data, we observe willful empiricism of the physician. Half of the medical records describe the outcomes of hospitalized people and indicate very high records of deaths, followed by referrals to other hospitalization institutions to prolong confinement.

**CONCLUSIONS:**

Internees suffered from isolation and assistance focused on state policy allied to science, especially psychiatry, to legitimize exclusion of the socially undesirable.

## INTRODUCTION

The history of psychiatry, with its knowledge and practices, is intrinsically correlated to the history and social life of cities, the scenarios in which this knowledge is built, developed, and strengthened^1^.

In São Paulo, during the second half of the 19th century, there were several changes in the political, economic, and social context. The city consolidated itself as a dynamic hub, the “coffee metropolis”, and presented important growth, progress, urbanization, and industrialization. In contrast, we have to consider the growing inequality, unfavorable conditions, and poverty of a significant part of the population that enjoyed very little this set of new introductions^2^.

An important portion of the population that was on the margin of the described improvements was that constituted by blacks recently freed from the servile regime^3^.

The context at the beginning of the 20^th^ century was rapid economic growth of the city, initially through coffee and later by industry, added to the full implementation of the project of a modern and orderly nation intended by the elites, led by the republicans of São Paulo^4^.

Economic growth brought with it significant demographic growth: the number of inhabitants between 1880 and 1920 was from 35,000 to 600,000. There was European immigration stimulated and favored by state policy, as well as migration to the cities of black people recently freed from slavery, who sought to move away from the old servile relations and find better job opportunities in the capital. However, São Paulo represented one of the cities least conducive to the absorption of black individuals recently freed from slavery^5,6^.

Theories of whitening were praised, and official state public policies were implemented in order to favor whiteness. The biological, intellectual, moral, and cultural superiority of white people was widespread among elite intellectuals and politicians. Interracial relations were considered an undesirable step, but necessary for whitening^7^.

The assumptions of whitening were used as justification for the permissiveness of violence, especially sexual violence, against black women^8^. The importation of European white labor was another resource used to reject and take employment from the national worker, since only European immigrants represented the progress and civilization worthy of the São Paulo society^7^.

Black people were denied opportunities for work, education, housing, and sanitation, unlike what happened with European immigrants. Benefits towards new businesses, tax incentives, bank loans, financial aid, opportunities for children as apprentices, were offered to these immigrants^7^.

The image linked to black people was of the personification of backwardness, underdevelopment, disqualification, laziness, indolence, incapacity and subject to elimination. In this scenario, those who were previously victims of physical deformities resulting from heavy overwork, bodily mutilations inherited from punishments and torture, had any possibility of integration curtailed^7,8^.

However, those considered maladjusted, in this new disposition, worried the social fragments that sought civilization. The idea of socially and morally reforming certain subjects, isolated in corrective spaces, seemed to be an interesting strategy for part of society^9^.

Simultaneously and opportunely, the medical thinking that was imposed as an instance of social control of people and populations, began the construction of a social medicine that acted on individuals and society. Brazilian psychiatry emerged as the specific, apparently necessary field of medicine to respond to the claims of different sectors of society in relation to madness and was based on some constructs of European theories of the time^10^.

The progress and important social influence that medicine exerted, was given through “hygiene” already in the first decades of the 20^th^ century, which incorporated the city and population to the field of medical knowledge. Hygienists inaugurated a movement that disciplined, hierarchized, and led to submission; through conservative views, it interfered in public and private places^2^.

In addition, between 1890 and 1920, ideas of the hierarchy of races acquired tones of scientific legitimacy. The Brazilian intellectual elite absorbed European deterministic theories and developed racial theories in Brazil, which preached white racial superiority^2^.

The first Brazilian eugenic organizations emerged in 1917, in São Paulo. The term “eugenics” meant the “science of racial enhancement”, and also structured the psychiatric knowledge of the period. Eugenicists aimed to remove the races considered less developed, responsible for “social ills”, and “rationalize natural selection”, in order to generate a more developed society^11^.

The concept of “degeneracy”, inaugurated by the European theorist Morel, represented “a deviation from the ideal type”, “the degradation of Customs“, “the gradual and progressive decay of a perfect primitive type”^12^. Degeneracies and deviations were seen as products of heredity and social disorder^13^.

Black individuals were considered inferior, so the formation of a “Brazilian nation“ and “São Paulo metropolis” was considered possible only from the disappearance of “inferior and savage” types. The European immigrant was instrumental in enabling population whitening and the formation of a selected racial type^14^.

Some other themes were incorporated into the setting of frontiers that would separate the boundaries of “normal“ and “pathological”. As examples, we can cite civilization, “race“, sexuality, work, “alcoholism”, delinquency/criminality, religion, and political contestation. Thus, the ”threats” to the established order and progress could be identified and associated with mental disorder^13^.

The Juquery Asylum, based on these theories, fulfilled the task of marginalizing those considered insane in places far from the center and subjecting them to the ideology of work and social control^2^.

By reading Michel Foucault^15^, Santos^16^ (2020) refers to the theory of state racism as a mechanism that makes it possible to legitimize the annulment of populations in a society. This conception comes from the description of biopower, a term that indicates the “social organization under the argument of maintaining the well-being of populations”, that is, the techniques of biopower aim to “make live and let die”. It is exercised by everyone, but the State is the one who controls some of its practices. Thus, the techniques of power will operate on individual bodies, disciplining them for work, making them obedient and skillful and sometimes punishing them, but also using new technologies aimed at multiplicity.

Medicine becomes one of the most important protagonists of this technology, since it creates a healthy population capable of producing and consuming. With its knowledge-power, medicine becomes a policy of intervention on the individual and collective, with disciplinary effects. Also from biopolitics, the right to kill arises, since the population becomes a scientific and political problem^15,16^. Thus, psychiatry was and is widely used for social intervention policy.

The nascent São Paulo psychiatry, through the asylum as the main instrument, legitimized the social exclusion of black people, in a society that had recently emerged from slavery and for which black people had no place. The psychiatric institution provided space for “treatment“ towards subjects who did not fit into the current social order and had important personalities for the effectiveness of this “cure”^9^.

Francisco Franco da Rocha was a central character in the process of constitution of Brazilian psychiatry. The first alienist in São Paulo and armed with full authority, he was assigned to guide the government in the reform of assistance to the alienated. Through arguments and scientific influence by writing articles in the press, as well as notable political influence, he was responsible for the creation and direction of the Juquery Asylum in 1898^17,9^.

The Juquery Asylum was one of the largest, most populous, oldest and for many decades considered the model asylum for Brazilian psychiatry. An institution inaugurated to meet the most up to date alienistic knowledge, promote innovative experiences, in addition to being able to solve in a disciplinary way the problems raised by the urban explosion. During the direction of Franco da Rocha, it had a mixed care model, composed of the asylum and agricultural colony. It associated chemical and mechanical therapies (mental medicine) with labor therapy and moral treatment (traditional asylum)^17^.

The installation of the Juquery Asylum responded to the appeals of the newly graduated Brazilian psychiatrists, so that the assistance to the so-called madness was based on scientific constructs of the flourishing psychiatry, at the same time that it served as a base for state policies towards exclusion and social hygiene, racial issues among which the whitening of the population stands out. It was the first of a series of asylums to be built in the country, but with the differential of having been born to be the source of irradiation of psychiatric knowledge and power.

In 1923, after Franco da Rocha and with wide prominence and influence in the medical, social, political, and academic spheres, Antonio Carlos Pacheco e Silva took over the direction of Juquery. During his administration, the organicist model associated with eugenics was strengthened, however, without abandoning the moral aspects. There was also a greater focus on biological therapies and anatomopathological studies. Pacheco e Silva was a member of the Brazilian league of Mental Hygiene and in 1926 founder of the São Paulo League of Mental Hygiene^18^.

It is important to highlight the conceptions of Franco da Rocha and Pacheco e Silva, since the scientific concepts of the directors in each period, closely linked to their political, cultural and economic conceptions, directly influenced the guidelines that the other psychiatrists who made up Juquery would follow^11^.

Thus, this study is justified by the need to make emerge from the vastness of the obscure asylum universe the “assistance” to which black people were subjected. Through the medical records, in which the alienist is an omnipresent voice, in the context in which medicine, especially psychiatry, exercised normalizing character, of social control, we aim to analyze the asylum practices to which these black people were subjected, under the prism of the alienist.

The research sought to reveal the care provided to black individuals hospitalized in the period from 1898 to 1930 in the Juquery Asylum, having considered the social context and the hegemony of medical knowledge of the time.

## METHODS

This is an exploratory-descriptive study with a qualitative approach, using documentary analysis. The research was carried out in the archive of the Historical and Cultural Heritage of the Juquery Hospital Complex, with a data collection period between the months of July and December 2019.

We analyzed the contents of medical records of black individuals who were hospitalized at Juquery from 1898, the opening date of the institution, until 1930.

The proposed period for closing the collection was considered so that in these 32 years of existence of the Juquery it would be possible to encompass the directions of Franco da Rocha and Pacheco e Silva, which were immensely representative and based on ideas that strongly influenced the psychiatric behaviors of the time and permeated for many years with reflections in the present. We seek to fully cover the first administration and part of the period of the second (1923–1937). Thus, the entire transition between the labor therapy of Franco da Rocha and the organicism of Pacheco e Silva was contemplated in this work.

The abolition of slavery brings the apparent “oblivion” of the “non-white” population from the history of the First Republic (1889–1930). Racism in this specific period assumed a different dynamic from the conventional one in Brazil. It was characterized by an ostensible racism, violent and expressed in the law^7^. These specificities contributed to our choosing of such a time interval for the research.

Some authors also consider that from 1930, the question of black individuals was approached in a less negative and aggressive way, although later it was resumed in a way that was quite distant from the apparent “racial democracy” referred to at the time. From that decade on, the themes were reworked in a way that was simultaneously ironic and critical. For the first time, black people went from being exclusively pathological and negative to being represented as a positive contribution to Brazil^2^.

The research was authorized by the Juquery Hospital Complex and approved by the Ethics and Research Committee of the Federal University of São Paulo.

The medical records were organized in boxes, ordered between men and women and according to the dates of entry of the individuals. We performed the reading of each of the medical records, the specific information was collected, and each record was photographed in its entirety for later analysis.

Inclusion criteria were: being black, brown, *caboclo, moreno* or *mulato*; being a man or woman aged from 15 years, the age at which they were admitted with adults. Four participants aged 14 years were included because they had relevant information for the research. As non-inclusion criteria, we considered the incomplete fulfillment of medical records or little pertinent information, which hindered meeting the objective of the study.

The instrument prepared by the researchers was used, considering the names used at the time and the following information from the medical records: initials of the name and surname, general or internal record, age, ethnic data, profession, marital status, religion, nationality, origin, date of entry, anamnesis of entry, physical and psychological examination, diagnostic hypothesis, treatments performed, complications, outcome, and motive.

The data analysis was performed according to the stages of document analysis, that is, prior investigation was followed by preliminary analysis, which is applied in the dimensions: context, author, authenticity, reliability, nature of the text, internal logic, and key concepts. The dimensions were gathered and allowed a coherent interpretation from the initial questioning^19^.

The psychiatric theoretical frameworks of the period of eugenics, mental hygiene, degeneration, hierarchy of races, heredity and organicism, which structured the concept of madness, were used for the analysis of the researched data. We also considered the use of the Juquery Asylum for social hygiene, in an alliance between the state and “science”, which aimed at maintaining power and hegemonic interests in times of transformations in the São Paulo atmosphere.

## RESULTS AND DISCUSSION

All the medical records of the period were seen, about 6,300 records of blacks and whites, of which approximately 1,400 were of blacks. As for the minority of internees being black, it is not shown as a contrast with the policy of the period, because we have to consider that part of the blacks did not arrive at the Juquery Asylum; they were rather taken to the prison system of the time (public prisons, central police, prison, military institution) and to Collection, a waiting space for a place in hospitalization. In the analysis of the origin of these Juquery inmates, we verified that the majority came from these institutions of control and repression. The Juquery Asylum and the prison system formed the basis for the social exclusion of blacks.

We emphasize that the first republican penal code, published in 1890, was characterized by a considerable mechanism of discipline for work and persecution of the popular strata, represented especially by the freed slaves, to the extent that it criminalized “*vadios* and *capoeiras*”^4^. Thus, the expectation is that those black people who did not arrive at the asylum were likely to find themselves in prisons.

The medical records of whites were viewed only with the intention of certifying that they were not black people, since some of them were identified as whites on the cover, however, in the descriptions of alienists they were identified as blacks.

A total of 457 medical records were included in the study, 140 of women and 317 of men, as they were considered to contain information for the study objectives.

The descriptions in each of the medical records were used for “ethnic data”, a term used in most of the medical records. Since 1929, they have also used the term “color”.

We highlight important diversity concerning classifications for ethnic/color data that meet the numerous and specific classifications addressed by Nina Rodrigues at the time. He distinguished the Brazilian population, mostly *mestiços*, in “very varied degrees of interbreeding”. He argued that the “mixture between very dissimilar races of men seemed to produce a worthless type of mind, which would be of no use at all to any kind of life.”^20^.

The Brazilian *mestiço* population, therefore, is classified in gradation ranging from totally unusable to a useful product, capable of some superior manifestation. Most Brazilian theorists of the time adhered to the concept of higher and lower races. Thus, we realized that the *mestiço* was classified through physical characteristics, skin color, so that, in some cases, those considered superior because they resembled whites, could cross the limits imposed by color and obtain some status, being reclassified as white, or very close to it^21^.

Apparently, from the classification contained in the item “ethnic color/data”, it was possible to assess the level of proximity or distance of the individual from the socially desirable white and, from this, the level of mental deterioration of the person^20^.

From so many categories for this item, we perceive the representation of much more ideological and sociopolitical content, than biological. Since the black population of the time represented for the elite a threat to “civilization”, race mixing represented the destitution of the numerical importance of blacks, diluted in the white population, making it difficult to build robust racial and ethnic identities in the scenario of those oppressed by the system^21^.

Regarding education, few medical records contained this information; most of them were illiterate or considered uneducated by alienists.

Access to knowledge can also be used as a strategy of domination, of power, which would be able to maintain the current thought of white superiority. Thus, the presence of black people in schools was no longer prohibited, but subtle mechanisms of discrimination would always be present and made it difficult for them to remain in this space, as ways to reinforce inequalities, which at that time were no longer demarcated by the status of “slave”^22^. Black people suffered disadvantages in access to better jobs and information as a result of difficult access to schooling. In relation to black women, literacy rates were even lower^23^.

Most of the medical records did not contain information about the places from which people came. Among those who had this datum, most were related to the prison system, with descriptions of involvement with the police or passages through the jail/police station and the collection.

Management of control of the social order supported by the current legislation, brought the conception of the ideology of criminalized loitering, which had as its main characteristic the surveillance and repression of police authorities in relation to black individuals. They translated the unemployment and public policies to protect this population as an option not to work, as moral fragility^24^.

Many of the black people judged as loiters who were collected or arrested were itinerant workers looking for work. The representation of black individuals as imbeciles, demented, or professionally deformed seems to have been idealized by a racist elite, with the intention of justifying their supposed incapacity for free work and the need for immigration^7^.

At the time, São Paulo was one of the cities least conducive to the absorption of individuals newly freed from slavery^3^. There was the exaltation of whitening theories in a substantial way, so that the official public policies of the state were implemented with a view to favoring whiteness. Theories that highlighted the biological, intellectual, moral and cultural superiority of white people were widespread among intellectuals and politicians of the Brazilian elite. Race mixing was considered an undesirable step, but necessary for the rise of whitening^7^.

The model of establishing the free market in the capital of São Paulo, structured through mass immigration, was essentially discriminatory. Blacks were deprived of the old labor relations, in which they occupied the position of the main productive agent of the country, and prevented from reaching new opportunities, repelled as free labor^7^.

Most medical records do not provide data on profession; many were identified as rural workers (men) and domestic workers (women).

The conditions were even more deleterious for black women, who occupied the lowest position in the social pyramid, since they were reserved for multiple oppression, mainly due to racism and sexism^25^.

Being born a woman in this historical and sociocultural context of São Paulo, in itself, was considered a disability. Those who, in addition to women, were black, suffered doubly. They were seen as carriers of double inferiority, closer to nature than to the human condition, animalized, treated as a natural contingency of the asylum^17^.

We also noticed that the pattern of sexual abuse of black women took on enormous proportions; it managed to resist the abolition of slavery and, often, did not manifest itself in such an ostentatious way. Alienists of the period in question repeatedly portrayed black women as promiscuous and immoral. Let us remember that racism encourages and encourages sexual violence; it feeds sexism, as white men were convinced that they could sexually abuse black women and go unpunished. Racism and sexism are even more strongly effective in the obsolete sophistry of victim-blaming, who, because they are seen as “prostitutes” and “easy women”, had delegitimized their complaints about the violence suffered^26^.

Regarding the length of stay, more than 70% of the medical records of the participants correspond to long-term hospitalizations, which configures their institutionalization.

The purpose of such long hospitalizations does not seem to be linked to the possibility of cure for the supposed illness or exercise of therapeutic potentials, but to segregation. People who remained institutionalized for 30, 40 and even 69 years were banned from any possibility of restitution of their lives as social subjects^27^.

They were considered deviant, dangerous, crazy, unwanted, and excluded from participation in relationships, sentenced to social death, since they had their lives crossed by hospitalization and their views forever limited to the walls of the asylum.

For most of the individuals we analyzed, life ended in prison and the freedom outside the walls capable of rescuing and reconquering one’s individuality never arrived.

Regarding the diagnostic hypotheses of hospitalized people, the most frequent diagnoses were dementia, alcoholism, madness/manic-depressive madness, imbecility, epilepsy, psychosis, and mental debility.

Some of the internees described by the alienists did not suffer from the mental faculties or did not have any mental disorders. Some of them would be physically ill, others would be maladjusted to the family bosom, which reinforces the powerful use of the hospice as a social corrector. Some medical records showed “loitering” as a factor that would justify hospitalization.

Some of the photographs and descriptions depict people already deceased, who would have arrived for hospitalization dead or in very serious condition. In one of the cases, there is mention of an individual who would have arrived dead at the asylum, under the escort of soldiers, which raises assumptions about the reasons that would have triggered such a death, considering the presence of the escort.

As previously mentioned, the classification model of diagnoses and behaviors used in Juquery portrays the concepts of the directors of each period. Thus, the other psychiatrists who made up the asylum followed the established model^11^.

According to] the classification model used by Franco da Rocha, a significant part of the participants in the medical records studied would be classified among those with the greatest impairment, “second class”, of impeded mental development, rudimentary, incompatible with coexistence and permanence in the social environment. The second class would correspond to the diseases of frankly degenerate brains, anomalies closer to the teratological domain than to the pathological one; they did not bring favorable prognoses, had a constitutive character and were chronic^28^.

During the direction of Pacheco e Silva, organicist theories were strengthened, without abandoning the moral aspects in the formulation of diagnoses and behaviors. Biological therapies gained greater focus, as well as anatomopathological studies^18^

From 1923, the psychiatric model that gained notoriety in scientific publications, defended by Pacheco and Silva, and which was present in the formulation of the medical records of internees, was the organicist model associated with eugenics, the “science of racial enhancement”^29^.

For Pacheco e Silva, based on Darwin’s theory of evolution, it was possible to carry out “natural selection” in a rational way: higher genetic formation would be possible from the promotion of healthier humans and the elimination of degenerates, since the “ills of society” came from the less developed races. Practices such as involuntary sterilization and compulsory hospitalization of the defined “degenerates” were carried out with the intention of containing groups considered unwanted in the formation of that society^29^.

From these descriptions, one has the impression that these subjects composed a niche of people with immutable, permanent diagnostic conditions, not amenable to therapy or possibilities of return to society. We verified terms that refer to degeneration, inferior degeneration, intellectual decay, idiocy, imbecility, terms cited many times among the diagnoses found, which reinforces that a significant part of the diagnoses fell under the category “inferior degeneration” and, therefore, “incurable”.

The theory of degeneration allowed medicine to exercise power over the subject and the population, in a context in which heredity, evolutionary paradigms and racism were central. Thus, to understand black Individuals as inferior, to attribute this characteristic as something inherent to them and immutable, would be conceptions capable of fostering the desire for the elimination of the other, with the intention of strengthening the self, the white men, those for whom the state has provided sufficient power to legitimize the extermination^15^.

A lot of medical records do not contain data on treatments, which explains important contradictions of this medical institution, which is extremely systematic regarding its documentation, in which treatments are not apparent. We highlight medical records that mention exclusively clinical treatments, which would not justify staying in a psychiatric institution.

As for the present data, most of the treatments were medicated or through the combination of different formulas, which portrays the biological character of the interventions of the time. Labor therapy and praxitherapy are also described practices and enunciate the disciplinary approach of therapy. Protein therapy, clinotherapy, insulin therapy, pyrotherapy, enemas, purgatives and exclusively clinical treatments are also present, not to mention the rotunda (special confinement ward).

The fact that there was no therapeutic proposal described in the main document of the institution reinforces the hypothesis that the intention to keep these subjects in the asylum did not occur for the purpose of care, but as a deposit of incurability, of those who were outside the current norm, of beings rendered unusable by society, the “waiting room of cemetery for homeless people”^30^.

For few of them there would be the possibility of transforming incurability into productive incurability, through a therapeutic technique paradoxically aimed at incurable individuals. The treatment modality used was labor therapy or praxitherapy. The therapeutic technique of labor was then instituted as the training for the work of those who were considered loiterers. There was no escape from the old shackles of slavery^30^.

The rotundas were also used in treatment, that is, strong disciplinary cells of individual isolation for punishment of disobedient people that functioned more for punishment than treatment. Individuals remained for many days in these places, in a very small space reminiscent of solitary confinement in prisons, without clothes due to alleged risk to their lives, in subhuman conditions of hygiene, food, sleep; not infrequently deaths occurred due to lack of care.

Pyrotherapy, i.e., the induction of febrile peaks through various techniques (inoculation of malaria agent, proteins, intravenous calcium), in some participants was used in a way that generates a reflection on the extent to which treatment was configured. The used form portrays a number of applications beyond the recommended, even in the face of no significant improvement for the participants. The use of this therapy brings introjected to it the intensely punitive conception and the experimental character, by the insistence of alienists in the repetition of this treatment even without signs of improvement, disregard for the suffering generated and even deaths secondary to these therapies.

“Therapeutic “ abuses can also be exemplified by the insulin therapy used. Excessive increases in insulin units were carried out and generated important negative effects such as deep coma, called “accidental”. There was repetitive use of this technique alternating with others, even with inexpressive or negative results.

We found excessive sessions of convulsion therapy with Cardiazol, although no improvement was noted in the condition of the participant who continued to suffer from the negative effects of treatment without therapeutic potential. The suffering was exacerbated by the effects of the medication, which commonly led to a sense of imminent death.

With this unstoppable willful empiricism, even with the mostly negative results and the failure to repeat the organicist treatments, we had individuals becoming devoid of autonomy and thus hostages to the institutional order.

As for the outcomes of hospitalized people, half of the medical records of the participants do not describe what the outcome was. When such information is present, there are many records of deaths, most of which without specification of the reasons or causes that triggered it.

There are records of discharges, however, few were considered cured or improved. In some of them there is withdrawal by a family member with mention of death shortly after, which leads us to reflect on the condition in which patients were at the time of withdrawal.

In one of the discharge cases, the so-called “nurse” who took care the patient expressed the desire to have her at home as a maid. In another case, the person was sent to the residence of another employee, which reinforces family distancing, the expropriation of the subject’s autonomy and individuality, and the repetition of slave practices.

There were frequent records among the discharges, which referred individuals to other institutions such as asylums, clinics, colonies, other psychiatric hospitals, in which they remained institutionalized.

The individuals who had their lives cut short by Juquery Asylum did not have their deaths justified or documented. They were deprived of their lives and deaths, which from the date of entry no longer belonged to them.

Few were discharged, since the purpose of what was carried out at Juquery was not care, treatment or cure, but rather a widely replicated experiment, even in the face of the apparent low efficacy and improvement of those who were subjected to these practices^11^.

When the reason for the deaths was described, those due to respiratory issues were more frequent and tuberculosis was frequently cited. Deaths from gastrointestinal issues, such as enteritis, dysentery, diarrhea, are also very present, followed by deaths from hemorrhages, especially cerebral ones.

We emphasize that people died from malnutrition, cachexia, and anemia, some of them died after episodes of aggression by other internees and after physical fights with the employees themselves; there was even a record of police inquiries in the presence of the director of the institution, after one of these deaths.

One of the individuals died from “accidental intoxication”, one of them died from a state of toxin poisoning after acute abdomen by foreign body, some died shortly after entry – one of them died six hours after entering the *rotunda*. In one of the autopsies described in the medical records, it was possible to show more than 40 cysticerci in the brain and countless in the chest; in another, more than 48 *ascaris lumbricoides* in the intestine, which demonstrates the poor conditions of hygiene and sanitation.

It was common for participants to pass away without evaluation of the alienists. Others died as a result of entirely preventable forms of death, which portrays the neglect of care, such as those due to malnutrition, anemia, cachexia, or gastrointestinal issues. The same goes for those that after autopsy showed the infestations by parasitoses, in quite advanced degrees. A cruel portrait of neglect.

It is possible to notice that blacks died in greater proportions and were cured in smaller proportions, compared to whites^9^. Among those people who died, many were quite young. We highlight the high rates of deaths among blacks for the institution that was considered to be a model.

The fact that many medical records do not have diverse data portrays the degree of social importance of these people from the perspective of the alienists^9^.

The lack of assistance was marked by racism, medical power, negligence, punishment, neglect and multiple forms of institutional violence. In this scenario, the techniques and procedures carried out (such as isolation, punitive treatments, strict discipline, compulsory work, rewards and relations of preference between certain doctors or employees and internees, relations of possession, subordination, vassalage, domestication and servitude to the alienists) had the function of making the medical character the “master of madness”: “the one who makes it appear in its truth, who makes explicit what was hidden and silent, who dominates it, appeases it and absorbs it, after having wisely unleashed it”^31^.

In the case of the black population, the relations of subservience and slavery were only transferred from the *senzalas* to the asylum pavilions, from the slaveholders to the alienists.

## FINAL CONSIDERATIONS

Psychiatric care in the post-abolitionist period at the Juquery Asylum was structured in racist and moralistic paradigms.

The state gave sovereignty to medicine and science (especially psychiatry) in order to legitimize the exclusion of the socially undesirable. Medicine, with its “knowledge-power”, intervened in individual and community aspects, in a disciplinary and regulatory way, to exclude those not adequate to the established norm. Scientific racism and diverse forms of institutional violence characterized the psychiatric care provided to hospitalized black people, and operated through moral and organicist scientific constructs, theories of whitening, and eugenic and hygienic ideas.
