# Supplementary material for: Assistance to black people in the Juquery asylum from 1898 to 1930
Source: Rev Saude Publica. 2022 Oct 7;56:90. doi: 10.11606/s1518-8787.2022056004305 (PMC9550161; doi:10.11606/s1518-8787.2022056004305)
Supplement: Translation

# Translation: A assistência ao negro na instituição asilar do Juquery de 1898 a 1930

**Authors:** Amanda Carolina Franciscatto Avezani, João Fernando Marcolan

**Affiliations:** I Brasil Universidade Federal de São Paulo. Escola Paulista de Enfermagem. Programa de Pós-Graduação em Enfermagem. São Paulo, SP, Brasil

**Keywords:** Pacientes Internados, Grupo com Ancestrais do Continente Africano, Racismo, Hospitais Psiquiátricos, história, Assistência à Saúde Mental

## Abstract

**OBJETIVO:**

Desvelar a assistência prestada aos indivíduos negros internados no período de 1898 a 1930 no Hospício do Juquery, considerados o contexto social e a hegemonia do saber médico da época.

**MÉTODOS:**

Estudo exploratório-descritivo, qualitativo, análise documental, em prontuários de indivíduos negros(as) internados(as) desde a abertura do Hospício do Juquery em 1898 até 1930. Recorte temporal abarcou direções institucionais específicas e contexto histórico, político, econômico e social vivenciado pela população negra. Realizado no Arquivo do Patrimônio Histórico-cultural do Complexo Hospitalar do Juquery, entre julho e dezembro de 2019. Utilizado instrumento com questões referentes a dados sociodemográficos, data e anamnese de entrada, exame físico e psíquico, hipótese diagnóstica, tratamentos realizados, intercorrências, desfecho e motivo. Análise realizada segundo etapas da análise documental e pautada nos referenciais teóricos psiquiátricos do período.

**RESULTADOS:**

Foram vistos todos os prontuários do período, cerca de 6.300, dos quais aproximadamente 1.400 eram de negros(as). Desses prontuários, foram incluídos 457, 140 de mulheres e 317 de homens, considerados com informações significativas para objetivos do estudo. Maioria dos participantes teve internações de longa permanência, cuja finalidade não pareceu estar atrelada à possibilidade de cura ou reinserção social, mas à segregação. A partir dos diagnósticos descritos, a impressão é que esses sujeitos compunham nicho com condições imutáveis, permanentes, não passíveis de terapêuticas que possibilitassem retorno à sociedade, exemplificada pela degeneração. Quantidade significativa dos prontuários não traz dados sobre tratamentos, o que reforça a hipótese de que eram mantidos internados não para proposta de cuidado, mas como um depósito da incurabilidade; quando trazem dados observamos empirismo voluntarioso do médico. Metade dos prontuários descreve os desfechos das pessoas internadas e apontam registros muito altos de mortes, seguidos de encaminhamentos para outras instituições de internação a prolongar a vida de confinamento.

**CONCLUSÕES:**

Internados sofreram com isolamento e assistência focados na política de Estado aliada à ciência, sobretudo psiquiátrica, para legitimar exclusão dos socialmente indesejáveis.

## INTRODUÇÃO

A história da psiquiatria, com seus saberes e práticas, está intrinsecamente correlacionada à história e vida social das cidades, cenários onde esses saberes são construídos, desenvolvidos e fortalecidos^1^.

Em São Paulo, durante a segunda metade do século XIX, ocorreram diversas mudanças no contexto político, econômico e social. A cidade consolidou-se como polo dinâmico, “metrópole do café”, e apresentou importante crescimento, progresso, urbanização e industrialização. Em contraponto, destacaram-se a crescente desigualdade, as condições desfavoráveis e a pobreza de parte significativa da população que pouco usufruía desse conjunto de novas introduções^2^.

Parcela importante da população que esteve à margem das melhorias descritas foi aquela constituída por negros(as) recém libertos(as) do regime servil^3^.

O contexto no início do século XX era de rápido crescimento econômico da capital, inicialmente por meio do café e posteriormente pela indústria, somado à execução plena do projeto de nação moderna e ordeira pretendido pelas elites, lideradas pelos republicanos de São Paulo^4^.

O crescimento econômico trouxe consigo o significativo crescimento demográfico: a quantidade de habitantes entre 1880 e 1920 foi de 35.000 para 600.000. Ocorreu a imigração europeia estimulada e favorecida pela política do Estado e a migração para as cidades de pessoas negras recentemente libertas da escravidão, que buscavam se afastar das antigas relações servis e encontrar oportunidades melhores de trabalho na capital. Porém, São Paulo representava uma das cidades menos propícias à absorção de negros(as) recém libertos(as) da escravidão^5,6^.

As teorias de branqueamento eram exaltadas e as políticas públicas oficiais do Estado foram implementadas para favorecer a branquitude. A superioridade biológica, intelectual, moral e cultural do branco era difundida entre os intelectuais e políticos da elite. A mestiçagem era considerada uma etapa indesejável, mas necessária para o branqueamento^7^.

Os pressupostos do branqueamento foram utilizados como justificativa para a permissividade da violência, principalmente sexual, contra as mulheres negras^8^. A importação de mão de obra branca europeia foi outro recurso utilizado na intenção de rejeitar e desempregar o trabalhador nacional, uma vez que somente os imigrantes europeus representavam o progresso e a civilização dignos da sociedade paulista^7^.

Aos negros e negras foram negadas oportunidades de trabalho, educação, moradia e saneamento, diferentemente do ocorrido com imigrantes europeus. Benefícios e facilidades para iniciar empreendimentos, incentivos fiscais, empréstimos bancários, auxílio financeiro, oportunidades às crianças como aprendizes de ofício, eram ofertadas a esses(as) imigrantes^7^.

A imagem vinculada ao(à) negro(a) era de personificação do atraso, subdesenvolvimento, desqualificação, preguiça, indolência, incapacidade e sujeito passível de eliminação. Nesse cenário, aqueles que anteriormente foram vítimas de deformações físicas resultantes do excesso de trabalho pesado, mutilações corporais herdadas das punições e torturas, tiveram cerceada qualquer possibilidade de integração^7,8^.

No entanto, os considerados desajustados, nessa nova disposição, preocupavam os fragmentos sociais que buscavam a civilização. A ideia de reformar social e moralmente determinados sujeitos, isolados em espaços corretivos, parecia constituir estratégia interessante para parte da sociedade^9^.

Simultânea e oportunamente, a medicina que se impunha como instância de controle social das pessoas e populações, iniciou a construção de uma medicina social que atuou sobre indivíduos e sociedade. A psiquiatria brasileira surgiu como o campo específico, aparentemente necessário da medicina, para responder aos reclamos de diferentes setores da sociedade em relação à loucura e se pautou em alguns construtos das teorias europeias da época^10^.

O progresso e importante influência social que a medicina exercia, se deu por meio da “higiene” já nas primeiras décadas do século XX, que incorporou a cidade e população ao campo do saber médico. Os higienistas inauguraram movimento que disciplinou, hierarquizou e levou à submissão; por meio de visões conservadoras interferiu em locais públicos e privados^2^.

Além disso, entre 1890 e 1920, ideias de hierarquização das raças adquiriram foros de legitimidade científica. A elite intelectual brasileira absorveu as teorias deterministas europeias e desenvolveu teorias raciais no Brasil, que pregavam a superioridade racial branca^2^.

As primeiras organizações eugênicas brasileiras surgiram em 1917, em São Paulo. O termo “eugenia” significou a “ciência do aprimoramento racial” e também estruturou o saber psiquiátrico do período. Os eugenistas visavam retirar as raças consideradas menos desenvolvidas, responsáveis pelas “mazelas sociais”, e “racionalizar a seleção natural”, com o intuito de gerar uma sociedade mais desenvolvida^11^.

O conceito de “degenerescência”, inaugurado pelo teórico europeu Morel, representava “um desvio do tipo ideal”, “a degradação dos costumes”, “a decadência gradual e progressiva de um tipo primitivo perfeito”^12^. As degenerescências e desvios foram vistos como produtos da hereditariedade e da desordem social^13^.

Negros(as) foram considerados(as) inferiores, então, a formação de uma “nação brasileira” e “metrópole paulista” foi considerada possível apenas a partir do desaparecimento de tipos “inferiores e selvagens”. O imigrante europeu foi fundamental para possibilitar o branqueamento populacional e a formação de um tipo racial selecionado^14^.

Alguns outros temas foram incorporados na fixação de fronteiras que separariam os limites do “normal” e “patológico”. Como exemplos, podemos citar a civilização, a “raça”, a sexualidade, o trabalho, o “alcoolismo”, a delinquência/criminalidade, a religião e a contestação política. Assim, as “ameaças” à ordem e ao progresso estabelecidos poderiam ser identificadas e associadas ao transtorno mental^13^.

O Hospício do Juquery, embasado nessas teorias, cumpria o encargo de marginalizar os considerados loucos em locais distantes do centro e submetê-los à ideologia de trabalho e controle social^2^.

Em leitura de Michel Foucault^15^, Santos^16^ (2020) faz referência à teoria do racismo de Estado como mecanismo que possibilita legitimar a anulação de populações de uma sociedade. Inicia tal concepção a partir da descrição do biopoder, termo que indica a “organização social sob o argumento de manter o bem-estar das populações”, ou seja, as técnicas do biopoder visam “fazer viver e deixar morrer”. Ele é exercido por todos, mas o Estado é quem controla algumas de suas práticas. Assim, as técnicas de poder vão operar nos corpos individuais, disciplinando para o trabalho, tornando-os obedientes e hábeis e por vezes os punindo, mas também utilizando novas tecnologias voltadas para a multiplicidade.

A medicina passa a ser um dos protagonistas mais importantes dessa tecnologia, pois ela cria uma população saudável capaz de produzir e consumir. Com o seu saber-poder, a medicina torna-se política de intervenção sobre o individual e coletivo, com efeitos disciplinares. Também a partir da biopolítica, surge o direito de matar, uma vez que a população passa a ser problema científico e político^15,16^. Assim, a psiquiatria foi e é amplamente usada para política de intervenção social.

A nascente psiquiatria paulista, por meio do hospício enquanto instrumento principal, legitimou a exclusão social do(a) negro(a), em uma sociedade que saíra recentemente da escravidão e para a qual o(a) negro(a) não tinha lugar. A instituição psiquiátrica conferia espaço de “tratamento” para sujeitos não enquadráveis na ordem social vigente e contou com importantes personalidades para a efetivação dessa “cura”^9^.

Francisco Franco da Rocha foi personagem central no processo de constituição da psiquiatria brasileira. O primeiro alienista em território paulistano e munido de toda autoridade, designado a orientar o governo na reforma da assistência aos alienados. Por meio de argumentos e influência científica pela escrita de artigos na imprensa, bem como notável influência política, foi responsável pela criação e direção do Hospício do Juquery em 1898^17,9^.

O Hospício do Juquery foi um dos maiores, mais populosos, antigos e durante muitas décadas considerado o hospício-modelo da psiquiatria brasileira. Uma instituição inaugurada para atender aos mais atualizados saberes alienistas, promover inovadoras experiências, além de ser capaz de resolver de maneira disciplinar as problemáticas suscitadas pela explosão urbana. Durante a direção de Franco da Rocha, contou com um modelo de assistência misto, composto pelo hospício e colônia agrícola. Associou terapias químicas e mecânicas (medicina mental) com a laborterapia e o tratamento moral (asilo tradicional)^17^.

A instalação do Hospício do Juquery atendeu aos apelos dos recém-formados psiquiatras brasileiros, para que a assistência à denominada loucura fosse feita baseada em construtos científicos da psiquiatria florescente, ao mesmo tempo que servia de lastro para políticas de Estado no sentido de exclusão e higienização social, questões raciais dentre as quais destaca-se o embranquecimento da população. Foi o primeiro de uma série de hospícios a serem construídos no país, mas com o diferencial de ter nascido para ser a fonte de irradiação do saber e poder psiquiátrico.

Em 1923, posteriormente a Franco da Rocha e com amplo destaque e influência nas esferas médica, social, política e acadêmica, Antonio Carlos Pacheco e Silva assumiu a direção do Juquery. Durante sua gestão, fortaleceu-se o modelo organicista associado à eugenia, porém, sem abandonar os aspectos morais. Houve também maior enfoque para as terapias biológicas e estudos anatomopatológicos. Pacheco e Silva foi membro da Liga Brasileira de Higiene Mental e em 1926 fundador da Liga Paulista de Higiene Mental^18^.

Importante destacar as concepções de Franco da Rocha e Pacheco e Silva, uma vez que os conceitos científicos dos diretores em cada período, intimamente ligados às suas concepções políticas, culturais e econômicas, influenciavam diretamente as diretrizes que os demais psiquiatras que compunham o Juquery seguiriam^11^.

Dessa forma, este estudo justifica-se pela necessidade de fazer emergir da vastidão do obscuro universo manicomial a “assistência” à qual as pessoas negras foram submetidas. Por meio dos prontuários, nos quais o alienista é voz onipresente, no contexto em que a medicina, especialmente a psiquiatria, exercia caráter normalizador, de controle social, almejamos analisar as práticas asilares às quais essas pessoas negras foram sujeitas, sob o prisma do alienista.

A pesquisa teve por objetivo desvelar a assistência prestada aos indivíduos negros(as) internados no período de 1898 a 1930 no Hospício do Juquery, considerados o contexto social e a hegemonia do saber médico da época.

## MÉTODOS

Trata-se de estudo exploratório-descritivo de abordagem qualitativa, com uso da análise documental. A pesquisa foi realizada no Arquivo do Patrimônio Histórico-Cultural do Complexo Hospitalar do Juquery, com período de coleta de dados entre os meses de julho e dezembro de 2019.

Foram analisados os conteúdos de prontuários de indivíduos negros(as) que estiveram internados(as) no Juquery desde 1898, data de abertura da instituição, até 1930.

A escolha pelo período proposto para encerramento da coleta, considerou que nesses 32 anos de existência do Juquery seria possível abarcar as direções de Franco da Rocha e Pacheco e Silva, imensamente representativas e fundamentadas por ideários que influenciaram vigorosamente as condutas psiquiátricas da época e continuaram permeados por muitos anos com reflexos na atualidade. Buscamos abranger integralmente a primeira gestão e parte do período da segunda (1923–1937). Assim, toda a transição ocorrida entre a laborterapia de Franco da Rocha e o organicismo de Pacheco e Silva foi contemplada neste trabalho.

A abolição da escravidão traz o aparente “esquecimento” da população “não-branca” da história da Primeira República (1889–1930). O racismo nesse período específico assumia dinâmica diversa da convencional no Brasil. Caracterizava-se por se tratar de um racismo ostensivo, violento e expresso na lei^7^. Tais especificidades contribuíram para que elegêssemos tal intervalo temporal para a pesquisa.

Alguns autores consideram também que a partir de 1930, abordava-se a questão do negro(a) de maneira menos negativa e agressiva, embora posteriormente tenha sido retomada de maneira bastante distanciada da aparente “democracia racial” referida na época. A partir dessa década, os temas foram reelaborados de forma simultaneamente irônica e crítica. O(a) negro(a) passava, pela primeira vez, de presença unicamente patológica e negativa a ser representado como contribuição positiva para o Brasil^2^.

A pesquisa foi autorizada pelo Complexo Hospitalar do Juquery e aprovada pelo Comitê de Ética e Pesquisa da Universidade Federal de São Paulo.

Os prontuários encontravam-se organizados em caixas, ordenadas entre homens e mulheres e conforme as datas de entrada dos indivíduos. Realizamos a leitura de cada um dos prontuários, foram coletadas as informações específicas e cada prontuário foi fotografado na íntegra para análise posterior.

Constituíram critérios de inclusão: ser negro(a), pardo(a), caboclo(a), moreno(a) ou mulato(a); ser homem ou mulher com idade a partir de 15 anos, idade na qual eram internados junto aos adultos. Houve quatro participantes com 14 anos incluídos por terem informações relevantes para a pesquisa. Como critérios de não inclusão consideramos o preenchimento incompleto dos prontuários ou poucas informações pertinentes, que prejudicavam atender ao objetivo do estudo.

Foi utilizado instrumento elaborado pelos pesquisadores, consideradas as denominações utilizadas na época e as seguintes informações dos prontuários: iniciais do nome e sobrenome, registro geral ou interno, idade, dados étnicos, profissão, estado civil, religião, nacionalidade, procedência, data de entrada, anamnese de entrada, exame físico e psíquico, hipótese diagnóstica, tratamentos realizados, intercorrências, desfecho e motivo.

A análise dos dados foi realizada segundo as etapas da análise documental, ou seja, aprofundamento prévio seguido pela análise preliminar, que se aplica nas dimensões: contexto, autor, autenticidade, confiabilidade, natureza do texto, lógica interna e conceitos-chave. As dimensões foram reunidas e possibilitaram a interpretação coerente a partir do questionamento inicial^19^.

Os referenciais teóricos psiquiátricos do período de eugenia, higiene mental, degeneração, hierarquização das raças, hereditariedade e organicismo, que estruturavam o conceito de loucura, foram utilizados para a análise dos dados pesquisados. Também considerou-se o uso que se fez do Hospício do Juquery para higienização social, numa aliança entre o Estado e “ciência”, que visava a manutenção do poder e interesses hegemônicos em tempos de transformações nos ares paulistas.

## RESULTADOS E DISCUSSÃO

Foram vistos todos os prontuários do período, cerca de 6.300 prontuários, de negros(as) e brancos(as), dos quais aproximadamente 1.400 eram de negros(as). Quanto à minoria dos internos serem negros, não se mostra como contraste com a política ensejada no período, pois temos que considerar que parte dos negros não chegava ao Hospício do Juquery, era levada ao sistema prisional da época (cadeias públicas, polícia central, prisão, instituição militar) e ao Recolhimento, local de aguardo de vaga para internação. Na análise da procedência desses internos do Juquery, verificamos que a maioria vinha dessas instituições de controle e repressão. O Hospício do Juquery e o sistema prisional formaram a base para a exclusão social dos(as) negros(as).

Ressaltamos que o primeiro código penal republicano, de 1890, foi caracterizado por considerável mecanismo de disciplinarização para o trabalho e de perseguição às camadas populares, representadas especialmente pelo escravo liberto, na medida em que criminalizava “os vadios e capoeiras”^4^. Assim, o esperado é que essas pessoas negras que não chegaram ao hospício provavelmente se encontrassem nas prisões.

Os prontuários de brancos(as) foram visualizados apenas na intenção de certificar de que não se tratava de pessoas negras, pois alguns deles foram identificados como brancos(as) na capa, porém, nas descrições do alienista eram identificados(as) como negros(as).

Foram incluídos no estudo 457 prontuários, 140 de mulheres e 317 de homens, por considerarmos conterem informações para os objetivos do estudo.

Foram utilizadas as descrições em cada um dos prontuários para “dados ethnicos”, termo utilizado na maior parte dos prontuários. A partir de 1929, fazem também uso do termo “cor”.

Evidenciamos importante diversidade concernente às classificações para dados ethnicos/cor que vão ao encontro das numerosas e específicas classificações abordadas por Nina Rodrigues à época. Distinguiu a população brasileira, majoritariamente de mestiços, em “graus muito variados de cruzamentos”. Defendia que a “mistura entre raças de homens muito dessemelhantes parecia produzir um tipo mental sem valor, que não prestaria enfim para gênero algum de vida”^20^.

A população mestiça brasileira, portanto, é classificada em gradação que vai desde totalmente inaproveitável a um produto útil, capaz de alguma manifestação superior. A maioria dos teóricos brasileiros da época aderiu ao conceito de raças superiores e inferiores. Assim, percebemos que o mestiço era classificado por meio das características físicas, cor da pele, de modo que, em alguns casos, os considerados superiores por se assemelharem aos brancos, podiam transpor os limites impostos pela cor e obter algum status, sendo reclassificados como brancos, ou muito próximo a isso^21^.

Aparentemente, da classificação contida no item “dados ethnicos/cor”, era possível taxar o nível de proximidade ou distanciamento do indivíduo do branco socialmente desejável e, a partir disso, o nível de deterioração mental da pessoa^20^.

A partir de tantas categorias para esse item, percebemos a representação de conteúdos muito mais ideológicos e sociopolíticos, que biológicos. Uma vez que a população negra da época representava para a elite uma ameaça à “civilização”, a miscigenação representou a destituição da importância numérica dos(as) negros(as), diluídos(as) na população branca, a dificultar a construção de identidades raciais e étnicas robustas no cenário dos oprimidos pelo sistema^21^.

Em relação à instrução, poucos prontuários traziam essa informação; a maioria deles era analfabeta ou considerada sem instrução alguma pelo alienista.

O acesso ao saber pode também ser utilizado como estratégia de dominação, de poder, que seria capaz de manter o pensamento vigente de superioridade branca. Assim, a presença dos(as) negros(as) na escola não era mais proibida, mas os mecanismos sutis de discriminação sempre estariam presentes e dificultavam a permanência deles nesse espaço, como formas de reforçar as desigualdades, que nesse momento não eram mais demarcadas pelo status de “escravo(a)”^22^. Negros(as) sofriam desvantagens no acesso a empregos melhores e à informação em decorrência do acesso dificultado à escolarização. Em relação às mulheres negras, as taxas de alfabetização eram ainda menores^23^.

Grande parte dos prontuários não trazia informações sobre os locais dos quais as pessoas eram procedentes. Dentre os que tinham esse dado, a maioria esteve relacionada ao sistema prisional, com descrições de envolvimento com a polícia ou passagens pela cadeia/delegacia e pelo Recolhimento.

Gestões de controle da ordem social sustentadas pela legislação vigente, traziam a concepção da ideologia da vadiagem criminalizada, que tinha como principal característica a vigilância e repressão das autoridades policiais em relação aos indivíduos negros(as). Traduziam a ausência de emprego e de políticas públicas de proteção a essa população como uma opção por não trabalhar, enquanto fragilidade moral^24^.

Muitas das pessoas negras julgadas como vagabundas e recolhidas, presas por vadiagem, se tratava de trabalhadores(as) itinerantes à procura de serviço. A representação do(a) negro(a) enquanto imbecil, demente, deformado(a) profissionalmente, parece ter sido idealizada por uma elite racista, na intenção de justificar a suposta incapacidade deles para o trabalho livre e a necessidade da imigração^7^.

Na época, São Paulo constituía uma das cidades menos propícias à absorção dos indivíduos recém libertos da escravidão^3^. Ocorria a exaltação das teorias de branqueamento de maneira substancial, de modo que as políticas públicas oficiais do Estado foram implementadas com vistas a favorecer a branquitude. Teorias que destacavam a superioridade biológica, intelectual, moral e cultural do branco, eram difundidas entre intelectuais e políticos da elite brasileira. A mestiçagem era considerada etapa indesejável, porém necessária para ascensão do branqueamento^7^.

O modelo de instauração do mercado livre na capital paulista, estruturado por meio da imigração em massa, foi essencialmente discriminatório. Negros(as) eram destituídos das antigas relações de trabalho, nas quais ocupavam a posição de principal agente produtivo do país, e impedidos de alcançar novas oportunidades, repelidos enquanto mão de obra livre^7^.

A maioria dos prontuários não traz dados sobre a profissão das pessoas, muitos foram identificados como trabalhadores rurais (homens) e trabalhadoras domésticas (mulheres).

As condições eram ainda mais deletérias para as mulheres negras, que ocupavam a posição mais baixa da pirâmide social, pois a elas foi reservada a múltipla opressão, principalmente em razão do racismo e do sexismo^25^.

Nascer mulher nessa conjuntura histórica e sociocultural de São Paulo, por si só, era considerada uma deficiência. Aquelas que além de mulheres, eram negras, sofreram duplamente. Eram vistas como portadoras de dupla inferioridade, mais próximas da natureza do que da condição humana, animalizadas, tratadas como uma contingência natural do hospício^17^.

Percebemos ainda que o padrão de abuso sexual das mulheres negras tomou proporções descomunais, conseguiu resistir à abolição da escravatura e, muitas vezes, não se manifestou de maneira tão ostensiva. Os alienistas do período em questão, repetidamente, retrataram as mulheres negras como promíscuas e imorais. Lembremos que o racismo encoraja e estimula a violência sexual, alimenta o sexismo, pois os homens brancos foram convencidos de que poderiam abusar sexualmente das mulheres negras e seguir impunes. O racismo e sexismo são ainda mais fortemente efetivados no obsoleto sofisma de culpar a vítima, que, por serem vistas como “prostitutas” e “mulheres fáceis”, tinham deslegitimadas suas denúncias sobre as violências sofridas^26^.

Em relação ao tempo de internação, mais de 70% dos prontuários dos participantes corresponde a internações de longa permanência, que configura a institucionalização deles.

A finalidade de internações tão longas não parece estar atrelada à possibilidade de cura para o suposto adoecimento ou exercício de potenciais terapêuticos, mas à segregação. Pessoas que permaneceram durante 30, 40 e até 69 anos institucionalizadas foram banidas de qualquer possibilidade de restituição de suas vidas enquanto sujeitos sociais^27^.

Foram considerados desviantes, perigosos, loucos, indesejados e excluídos da participação nas relações, condenados à morte social, uma vez que tiveram suas vidas atravessadas pela internação e suas vistas limitadas para sempre às paredes do hospício.

Para a maioria dos indivíduos que analisamos, a vida se encerrou no cárcere e a liberdade extramuros capaz de resgatar e reconquistar a própria individualidade, nunca chegou.

Referente às hipóteses diagnósticas das pessoas internadas, foram mais frequentes diagnósticos de demência, alcoolismo, loucura/loucura maníaco-depressiva, imbecilidade, epilepsia, psicose e debilidade mental.

Alguns dos internados descritos pelos alienistas não sofriam das faculdades mentais ou não tinham quaisquer perturbações mentais. Alguns deles estariam fisicamente doentes, outros seriam inadaptados ao seio familiar, o que reforça o potente uso do hospício enquanto corretor social. Alguns prontuários traziam “vagabundagem” enquanto fator que justificaria a internação.

Algumas das fotografias e descrições retratam pessoas já falecidas, que teriam chegado para a internação mortas ou em estado muito grave. Em um dos casos, há menção a indivíduo que teria chegado morto ao hospício, sob escolta de soldados, o que levanta suposições sobre os motivos que teriam desencadeado tal morte, considerada a presença da escolta.

Conforme citado anteriormente, o modelo de classificação dos diagnósticos e condutas utilizado no Juquery, retrata os conceitos dos diretores de cada período. Assim, os demais psiquiatras que compunham o hospício seguiam o modelo instituído^11^.

De acordo com o modelo de classificação utilizado por Franco da Rocha, parte significativa dos participantes dos prontuários estudados estaria classificada entre aquelas com maior prejuízo, “segunda classe”, de desenvolvimento mental impedido, rudimentar, incompatível com a convivência e permanência no meio social. A segunda classe corresponderia às moléstias dos cérebros francamente degenerados, anomalias mais próximas do domínio teratológico do que do patológico; não traziam prognósticos favoráveis, tinham caráter constitutivo e eram crônicas^28^.

Durante a direção de Pacheco e Silva, fortaleceu-se as teorias organicistas, sem abandonar os aspectos morais na formulação dos diagnósticos e condutas. As terapias biológicas ganham maior enfoque, bem como estudos anatomopatológicos^18^.

A partir de 1923, o modelo psiquiátrico que ganhou notoriedade nas publicações científicas, defendido por Pacheco e Silva, e que se fazia presente na formulação dos prontuários médicos dos internados, era o modelo organicista associado à eugenia, “ciência do aprimoramento racial”^29^.

Para Pacheco e Silva, embasado na teoria da evolução de Darwin, era possível realizar a “seleção natural” de forma racional: a formação genética superior seria possível a partir da promoção de humanos mais sadios e da eliminação dos degenerados, pois as “mazelas da sociedade” eram provenientes das raças menos desenvolvidas. Práticas como a esterilização involuntária e internação compulsória dos definidos “degenerados” eram realizadas na intenção de conter grupos considerados indesejados na formação daquela sociedade^29^.

A partir dessas descrições, tem-se a impressão de que esses sujeitos compunham um nicho de pessoas com condições diagnósticas imutáveis, permanentes, não passíveis de terapêuticas ou possibilidades de retorno à sociedade. Verificamos termos que dizem respeito à degeneração, degeneração inferior, decadência intelectual, idiotia, imbecilidade, termos citados muitas vezes entre os diagnósticos encontrados, o que reforça que parte significativa dos diagnósticos se enquadrava no quesito “degeneração inferior” e, portanto, “incurável”.

A teoria da degeneração permitiu à medicina exercer o poder sobre o sujeito e a população, em contexto no qual a hereditariedade, paradigmas evolucionistas e racismo eram centrais. Assim, taxar o indivíduo negro(a) enquanto inferior, atribuir essa característica como algo inerente a ele e imutável, seriam concepções capazes de fomentar o desejo de eliminação do outro, na intenção de fortalecer a si, homem branco, aqueles para os quais o Estado propiciou poder suficiente para legitimar o extermínio^15^.

Quantidade extremamente significativa dos prontuários não traz dados sobre tratamentos, o que explicita contradições importantes dessa instituição médica, extremamente sistemática no que diz respeito às suas documentações, nas quais os tratamentos não se fazem aparentes. Destacamos prontuários que fazem menção exclusivamente a tratamentos clínicos, o que não justificaria a permanência numa instituição psiquiátrica.

Quanto aos dados presentes, a maior parte dos tratamentos era medicamentoso ou por meio da combinação de fórmulas diversas, o que retrata o caráter biológico das intervenções da época. A laborterapia e praxiterapia também são práticas descritas e enunciam o enfoque disciplinador da terapêutica. Se fazem também presentes a proteinoterapia, clinoterapia, insulinoterapia, piretoterapia, clisteres, purgativos e tratamentos exclusivamente clínicos, sem deixar de citar a rotunda.

O fato de não haver proposta terapêutica descrita no principal documento da instituição reforça a hipótese de que a intenção em manter esses sujeitos no hospício não ocorria para a proposta de cuidado, e sim enquanto depósito da incurabilidade, dos que se encontravam fora da norma vigente, dos seres inutilizados pela sociedade, a “sala de espera dos cemitérios de indigentes”^30^.

Para poucos deles haveria a possibilidade de transformar a incurabilidade em incurabilidade produtiva, por meio de uma técnica terapêutica destinada paradoxalmente a indivíduos incuráveis. A modalidade de tratamento empregada a eles foi a laborterapia ou praxiterapia. Estava então instituída a técnica terapêutica de emprego do trabalho como o adestramento para o trabalho daqueles que eram considerados vagabundos. Não havia escape dos velhos grilhões da escravidão^30^.

Eram utilizadas nos tratamentos também as rotundas, isto é, celas fortes disciplinares de isolamento individual para punição de desobedientes que funcionavam mais para castigo do que tratamento. Comumente as pessoas permaneciam durante muitos dias nesses locais, em espaço bastante reduzido que lembra as solitárias das prisões, sem roupas devido a alegado risco à sua vida, em condições subumanas de higiene, alimentação, sono; não raro ocorriam óbitos por ausência de cuidados.

A piretoterapia, indução de picos febris por meio de diversas técnicas (inoculação de agente da malária, de proteínas, cálcio endovenoso), em alguns participantes foi utilizada de maneira que gera uma reflexão sobre até que ponto configurou tratamento. A forma empregada retrata número de aplicações além do recomendado, mesmo diante da não observada melhora significativa para os participantes. O uso dessa terapêutica traz introjetada a concepção intensamente punitiva e o caráter experimental, pela insistência do alienista na repetição desse tratamento mesmo sem indícios de melhora, desconsideração pelo sofrimento gerado e até mesmo mortes secundárias a essas terapêuticas.

Os abusos “terapêuticos” podem ser exemplificados também pela insulinoterapia utilizada. Aumentos excessivos nas unidades de insulina foram realizados e geraram efeitos negativos importantes, como coma profundo, denominados “acidentais”. Ocorreu uso repetitivo dessa técnica alternada com outras técnicas diversas, mesmo com resultados inexpressivos ou negativos.

Evidenciamos excessivas sessões de convulsoterapia pelo cardiazol, embora não fosse notada melhora no quadro da participante que continuou a sofrer pelos efeitos negativos de tratamento que não apresentava potencial terapêutico para seu caso. Era comum o sofrimento exacerbado pela aura cardiazólica com sensação de morte iminente.

Com esse empirismo voluntarioso que não arrefecia, mesmo com os resultados majoritariamente negativos e o fracasso da repetição dos tratamentos organicistas, tínhamos o indivíduo desprovido de autonomia e refém da ordem institucional.

Quanto aos desfechos das pessoas internadas, metade dos prontuários dos participantes não descreve qual foi o desfecho, conclusões do caso. Quando tal informação se faz presente são muito altos os registros de mortes, para a maioria das quais não há especificação dos motivos ou causas que a desencadearam.

Há registros de altas, no entanto, poucos foram considerados curados ou melhorados. Em alguns deles há retirada por algum familiar e há menção de retirada e morte pouco tempo depois, o que nos leva a refletir sobre a condição na qual se encontrava no momento de retirada da instituição.

Em um dos casos de alta, a denominada “enfermeira” que estimava a paciente manifestou o desejo de tê-la em casa como empregada. Em outro caso, a pessoa foi encaminhada para a residência de outro funcionário, o que reforça o distanciamento familiar, a desapropriação da autonomia e individualidade do sujeito e a repetição das práticas escravistas.

Eram frequentes registros denominados altas, que encaminhavam os indivíduos para outras instituições como asilos, clínicas, colônias, outros hospícios, onde permaneciam institucionalizadas.

Os indivíduos que tiveram suas vidas atravessadas pelo Hospício do Juquery não tiveram sequer suas mortes justificadas ou documentadas. Foram destituídos de suas vidas e mortes, que a partir da data de entrada não mais lhes pertenciam.

Poucos receberam alta, posto que a finalidade do que foi realizado no Juquery não consistiu em cuidado, tratamento ou cura, mas sim em experimento de ampla replicação, mesmo diante da aparente baixa eficácia e melhora daqueles que eram submetidos a essas práticas^11^.

Quando descrito o motivo das mortes, as por questões respiratórias eram mais frequentes e a tuberculose foi bastante citada. As mortes por questões gastrointestinais, como enterites, disenterias, diarreias, também se fazem muito presentes, seguidas pelas mortes por hemorragias, principalmente as cerebrais.

Ressaltamos que pessoas morriam por desnutrição, caquexia e anemia, algumas delas faleciam após episódios de agressão por outros internos e após lutas corporais com os próprios funcionários; havia registro inclusive de inquéritos policiais na presença do diretor da instituição, posteriores a um desses falecimentos.

Um dos indivíduos faleceu após “intoxicação acidental”, um deles faleceu por estado toxêmico após abdome agudo por corpo estranho, alguns faleciam logo após a entrada – um deles faleceu seis horas após entrada na rotunda. Em uma das autópsias descritas em prontuário, foi possível evidenciar mais de 40 cisticercos no cérebro e incontáveis no tórax; em outra, mais de 48 *ascaris lumbricoides* no intestino, o que demonstra as condições precárias de higiene e saneamento.

Era comum os participantes falecerem sem avaliação do alienista. Outros faleciam em decorrência de mortes totalmente evitáveis, que retratam a negligência da assistência, como as por desnutrição, anemia, caquexia ou questões gastrointestinais. O mesmo vale para as que após autópsia demonstraram as infestações por parasitoses, em graus bastante avançados. Retrato cruel da desassistência.

É possível notar que os(as) negros(as) faleciam em proporções maiores e saíam curados(as) em proporções menores, comparativamente aos brancos^9^. Entre essas pessoas que morreram, muitas eram bastante jovens. Destacamos altas taxas de mortes entre os(as) negros(as) para instituição considerada modelar.

O fato de muitos prontuários não possuírem diversos dados retrata o grau de importância social dessas pessoas sob a ótica do alienista^9^.

A desassistência foi marcada por racismo, poder médico, negligência, punição, descaso e múltiplas violências institucionais. Nesse cenário, técnicas e procedimentos efetuados como isolamento, tratamentos punitivos, disciplina rigorosa, trabalho obrigatório, recompensas e relações de preferência entre certos médicos ou funcionários e internados, relações de posse, subordinação, de vassalagem, de domesticação e servidão ao alienista, tiveram por função fazer do personagem médico o “mestre da loucura”: “aquele que a faz aparecer em sua verdade, que explicita o que estava escondido e silencioso, que a domina, a apazigua e a absorve, depois de havê-la sabiamente desencadeado”^31^.

No caso da população negra, as relações de subserviência e escravidão foram apenas transferidas das senzalas aos pavilhões do hospício, dos senhores de escravos aos alienistas.

## CONSIDERAÇÕES FINAIS

A assistência psiquiátrica no período pós-abolicionista no Hospício do Juquery foi estruturada em paradigmas racistas e moralistas.

O Estado propiciou à medicina e ciência, sobretudo à psiquiatria, soberania para legitimar a exclusão dos socialmente indesejáveis. A medicina, com o “saber-poder”, interviu nos aspectos individuais e comunitários, de forma disciplinar e regulamentadora, para excluir os não adequados à norma instaurada. O racismo científico e diversificadas formas de violência institucional caracterizaram a assistência psiquiátrica prestada às pessoas negras internadas e operaram por meio de construtos científicos morais e organicistas, teorias de branqueamento, ideários eugênicos e higiênicos.
